# CAR-T cell therapy for hepatocellular carcinoma: current trends and challenges

**DOI:** 10.3389/fimmu.2024.1489649

**Published:** 2024-11-06

**Authors:** Yexin Zhou, Shanshan Wei, Menghui Xu, Xinhui Wu, Wenbo Dou, Huakang Li, Zhonglin Zhang, Shuo Zhang

**Affiliations:** ^1^ Hospital of Chengdu University of Traditional Chinese Medicine, Chengdu, Sichuan, China; ^2^ The General Hospital of Western Theater Command, Chengdu, China; ^3^ Department of Radiation Oncology, Radiation Oncology Key Laboratory of Sichuan Province, Sichuan Clinical Research Center for Cancer, Sichuan Cancer Hospital & Institute, Sichuan Cancer Center, Affiliated Cancer Hospital of University of Electronic Science and Technology of China, Chengdu, Sichuan, China

**Keywords:** chimeric antigen receptor T cell, hepatocellular carcinoma, antigen, gene therapy, immunotherapy

## Abstract

Hepatocellular carcinoma (HCC) ranks among the most prevalent cancers worldwide, highlighting the urgent need for improved diagnostic and therapeutic methodologies. The standard treatment regimen generally involves surgical intervention followed by systemic therapies; however, the median survival rates for patients remain unsatisfactory. Chimeric antigen receptor (CAR) T-cell therapy has emerged as a pivotal advancement in cancer treatment. Both clinical and preclinical studies emphasize the notable efficacy of CAR T cells in targeting HCC. Various molecules, such as GPC3, c-Met, and NKG2D, show significant promise as potential immunotherapeutic targets in liver cancer. Despite this, employing CAR T cells to treat solid tumors like HCC poses considerable challenges within the discipline. Numerous innovations have significant potential to enhance the efficacy of CAR T-cell therapy for HCC, including improvements in T cell trafficking, strategies to counteract the immunosuppressive tumor microenvironment, and enhanced safety protocols. Ongoing efforts to discover therapeutic targets for CAR T cells highlight the need for the development of more practical manufacturing strategies for CAR-modified cells. This review synthesizes recent findings and clinical advancements in the use of CAR T-cell therapies for HCC treatment. We elucidate the therapeutic benefits of CAR T cells in HCC and identify the primary barriers to their broader application. Our analysis aims to provide a comprehensive overview of the current status and future prospects of CAR T-cell immunotherapy for HCC.

## 1 Introduction

Hepatocellular carcinoma (HCC) poses a significant global health challenge and ranks as the third most common cause of cancer-related mortality worldwide (1). While surgical resection and local ablation continue to serve as the mainstay treatment options for early-stage HCC patients, the utilization of diverse systemic therapies has become indispensable in enhancing prognosis for individuals in intermediate to advanced stages (2, 3). But current therapeutic options are seldom curative and the prognosis of patients with HCC remains grim (2, 3). Therefore, the exploration of innovative and efficient therapeutic strategies holds the utmost importance.

Recently, immunotherapy has opened up the possibility of new scenarios for treating advanced HCC (4). The immune system’s access to the liver is tightly regulated, and the liver’s immunosuppressive environment has evolved to defend against immune attacks (5, 6). As HCC typically expresses identifiable tumor-associated antigens, such as alpha-fetoprotein (AFP), glypican-3 (GPC3), or cellular-mesenchymal epithelial transition factor (c-MET) (7), there is a strategic opportunity where treatments could potentially stimulate or augment an anti-tumor immune response through vaccination or targeted therapeutic interventions (8, 9). Immune checkpoint inhibitors have been previously successfully employed in clinical settings for the management of HCC (10). On the contrary, chimeric antigen receptor (CAR) T cell therapy has shown significant therapeutic efficacy in patients with hematological malignancies (11, 12); nevertheless, further optimization of the technology is required for the effective and safe therapy of solid tumors including HCC (13, 14). The extensive research efforts by multiple investigators in both preclinical and clinical settings to assess CAR-T therapy against HCC underline a paradigm shift in the treatment (15). This review emphasizes the potential of applying CAR-T cells in HCC therapy, discussing aspects related to their design and delivery, recent therapeutic advancements, encountered challenges, and potential solutions.

## 2 Basics of CAR-T cell immunotherapy

**Figure 1:**
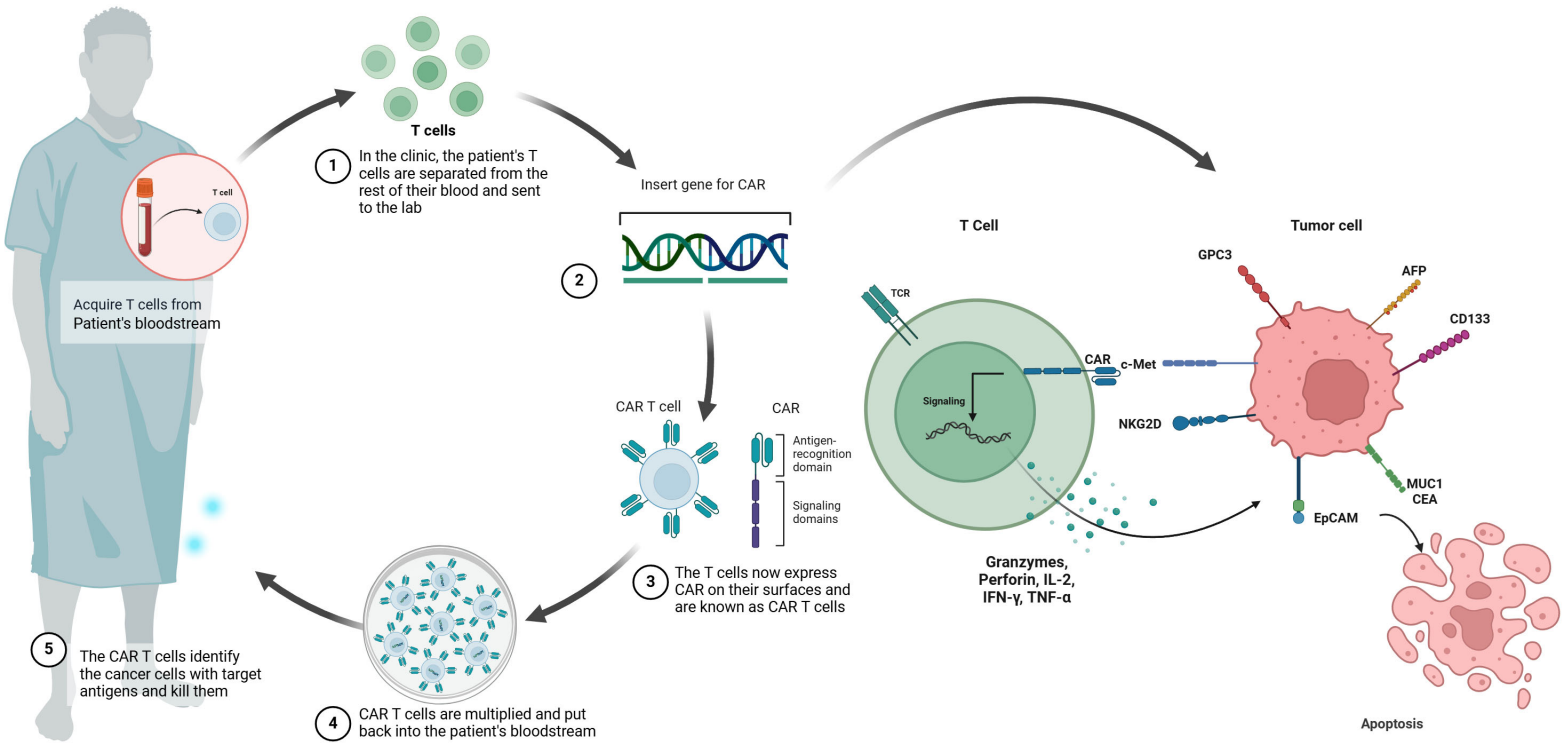
Schematic illustration of CAR-T cell therapy in HCC. chimeric antigen receptor (CAR) T cell therapy involves the initial extraction of T cells from the patient’s peripheral blood, followed by the introduction of a CAR gene into the T cells using a viral vector. This process results in the production of CAR T cells. After the CAR T cells are multiplied *in vitro*, they undergo a thorough assessment for cellular quality and are then aseptically filled. The final step involves administering the CAR T cells to the patient, where they are able to selectively bind to molecular targets such as GPC3, AFP, NKG2D, CD133, EpCAM, MUC1, CEA and c-MET.

The fundamental concept behind CAR-T cell immunotherapy is to combine the strength of a T cell with the targeting accuracy of an antibody to recognize specific tumor antigens (11). CAR-T therapy aims to introduce specific CARs into T cells in a relatively short timeframe (15). Expansion of these engineered T cells leads to the generation of memory and effector lymphocytes with high affinity *in vitro* (14, 15). Subsequently, these T cells are reinfused into the patient to undergo robust proliferation (14). The engineered CAR provides specificity, while the intracellular signaling domains trigger T cell-mediated cytotoxicity for eliminating cancer cells regardless of major histocompatibility complex (MHC) presentation (16, 17). CAR-T cells have been referred to as ‘living medications’ due to their ability to undergo proliferation and differentiation into durable memory cells, therefore inducing specific and enduring anti-tumor immune responses (11, 17) (**Figure 1**).

When viewed structurally, CARs can be divided into four distinctive components (18). There exists the antigen-binding domain and a linker region in the extracellular part of CARs (18). The antigen-binding domain typically derived from a single-chain variable fragment (scFv) commonly found in antibodies, allowing the CAR to attach to the target antigen present on the tumor cells (19, 20). Following this section comes a spacer or hinge region, which is subsequently linked to a transmembrane domain (21). Finally, an intracellular domain is present to convey signals to the CAR-modified cells (22, 23).

This intracellular domain can be further categorized into costimulatory domains and signaling domains (24, 25). Specific regions of CD28, 4-1BB, ICOS, or OX40 are commonly utilized as co-stimulatory domains (24–26). The CD28 co-stimulatory receptor on T cells plays a vital role in transmitting essential signals for continuous signaling, cell growth, and preventing immune exhaustion (27). Meanwhile, 4-1BB and OX40 act as co-stimulatory receptors impacting T cell activation, maturation, and apoptosis induction (28, 29). Overall, the integration of these co-stimulatory domains has proven to enhance the effectiveness of CAR-T cells in terms of cytokine secretion, T cell expansion, proliferation, and differentiation (30, 31). CAR-T cell generations are broadly classified based on the arrangement of their intracellular signaling domains (32). The first generation of CAR consists only of the CD3ζ signaling domain, with the addition of extra co-stimulatory signaling domains in the second generation (33, 34). In the third generation, two co-stimulatory domains are combined (35). Moreover, introducing a single co-stimulatory structural domain, in conjunction with another transgene, can be utilized to amplify cytokine production and thereby strengthen the functionality of CAR-T cells in fourth-generation CAR-T cell therapy (36). Contrasting with previous generations, the fifth generation of CAR-T cells includes an additional intracellular domain (37). Full activation of T cells is exclusively attained when both CARs engage simultaneously with their corresponding target antigens expressed on the cells (14, 18). CAR-T cells will not be activated by normal cells expressing only one of the two target antigens, thus evading elimination (38).

Both clinical and preclinical evidence have highlighted the substantial roles each of these regions plays in the overall functionality of CAR-T cells (39). The design of CARs is instrumental in determining the attributes of the associated CAR-T cells, encompassing elements such as antigen specificity, activation capability, cytotoxicity, proliferation potential, expansion capacity, persistence, and safety profile (40, 41). Therefore, it is imperative to select the optimal CAR format tailored to the specific needs of individual applications.

## 3 Preclinical and clinical evidence of CAR-T cells against HCC

Compared to conventional antitumor pharmaceuticals, CAR-T cell therapy exhibits distinct characteristics (41). This novel therapeutic approach represents a precision-targeted strategy for the management of neoplastic growths (41). An ideal target antigen for CAR-T cell therapy in cancers should display elevated levels of expression on tumor cells while showing either insignificant or minimal expression on normal cells (42). Key target antigens investigated in preclinical studies and clinical trials encompass Mucin 1, GPC3, AFP, NK group 2D ligand (NKG2DL) and c-MET (**Table 1**).

**Table 1:** Current targets of CAR-T cell therapy in HCC.

Target	CAR construct	Mechanism	Reference
GPC3	GPC3-367-specific CAR	Eradicated GPC3-expressing HCC cells and hindered the HCC progression	(49)
GPC3	GPC3-specific CAR	Eradicated GPC3-positive HCC cells and sorafenib in conjunction with GPC3-targeted CAR-T cells has also shown effectiveness	(52–55)
GPC3	GPC3-targeted CAR-T cells expressing GLUT1 or AGK	The upregulation of GLUT1 or AGK conferred protection to the CAR-T cells against apoptosis, which significantly eradicated GPC3-expressing HCC cells	(56)
GPC3	IL-7 and CCL19-secreting GPC3-targeted CAR-T cells	GPC3-CAR-T cells were engineered to express IL-7 and CCL9 for stimulating the proliferation and facilitating migration, which effectively eradicated the tumor.	(44, 57)
GPC3	Bispecific CAR-T cells targeting FAP and GPC3	Engineered bispecific CAR-T cells targeting both FAP and GPC3 to address tumor diversity in HCC	(58)
AFP	AFP-targeted CAR	Bound to AFP peptides presented by HLA-A02:01 on tumor cells	(59, 60)
c-Met	Bispecific c-Met/PD-L1 CAR-T Cells	The bispecific CAR-T cells that target both c-Met and PD-L1 and showed notable cytotoxicity against c-Met^+^PD-L1^+^ HCC cells	(65)
c-Met	MET-CAR.CD28ζ	Recognized and eliminated HCC cells based on overall MET expression	(66)
NKG2D	NKG2D CAR-T	Recognized NKG2D ligands, triggering immune responses to inhibit tumor	(72)
NKG2D	NKG2D-BBz CAR	Recognized NKG2D ligands, triggering immune responses to inhibit tumor	(73)
CD133	CD133-specific CAR	Targeted delivery of a PD-1-blocking scFv by CD133-specific CAR-T cells that enhanced antitumour efficacy in HCC	(81)
CD133	CoG133-CAR	Dual antigen-binding capabilities targeting CD133 and GPC3 that ignificant eradication of HCC tumors	(82)
EpCAM	EpCAM-specific CAR	Targeted EpCAM for inhibiting tumor growth	(85)
MUC1	MUC1-specific CAR	Targeted MUC1 for inhibiting tumor growth	(89)
CEA	CEA-specific CAR	Targeted CEA for inhibiting tumor growth	(90, 91)

### 3.1 GPC3

GPC3 is an embryonic glycoprotein that is tethered to the cellular membrane via a glycophosphatidylinositol anchor, which has been demonstrated upregulated expression in various malignancies, notably HCC (43, 44). Being an oncofetal antigen, GPC3 exhibits high expression in over 70% of HCC cases (43). Emerging evidence has revealed that GPC3 exerts remarkably impacts on HCC progression (43). GPC3’s core protein interacts with Frizzled, the Wnt receptor, which results in amplifying Wnt/β-catenin cascade and elevated cell growth in HCC (45, 46). Moreover, GPC3 could be oncogenic activated by zinc-fingers and homeoboxes 2 (ZHX2) and C-myc, therefore promoting cell proliferation and differentiation in the setting of HCC (47, 48). Remarkably, Dargel and co-workers identified an HLA-A2-restricted peptide (GPC3-367) and employed peptide multimers to isolate GPC3-specific T cells; in this research, primary CD8+ T cells expressing the transgenic T-cell receptor recognizing GPC3 with specificity were identified. These T cells exhibited the capability to eradicate GPC3-expressing hepatoma cells *in vitro* and hinder the progression of HCC xenograft tumors in mice (49). Therefore, targeting GPC3 may be a promising strategy against HCC.

Existing evidence has mentioned that GPC3 seems to have a stronger connection with the utilization of CAR T cell therapy (50, 51). GPC3-specific CAR-T cells have demonstrated the capability to eradicate GPC3-positive HCC cells in laboratory settings and GPC3-positive HCC tumor xenografts in murine models (52, 53). The synergistic use of sorafenib in conjunction with GPC3-targeted CAR-T cells has also shown effectiveness (54, 55).

To improve therapeutic effectiveness, Sun et al. engineered GPC3-targeted CAR-T cells that overexpressed glucose transporter type 1 (GLUT1) or acylglycerol kinase (AGK) for the treatment of HCC (56). These engineered CAR-T cells demonstrated targeted and efficient elimination of GPC3-positive tumor cells *in vitro*, showcasing enhanced antitumor efficacy in comparison to the second-generation CAR-T cells (56). Additionally, the upregulation of GLUT1 or AGK conferred protection to the CAR-T cells against apoptosis following repeated encounters with tumor cells (56). In line with this, novel GPC3-CAR-T cells were engineered to express IL-7 and CCL9 for stimulating the proliferation and facilitating migration (44, 57). Significantly, in a phase I clinical trial, these modified CAR-T cells effectively eradicated the tumor upon intra-tumor administration in a patient with advanced GPC3-positive HCC (57). Zhou et al. engineered bispecific CAR-T cells targeting both fibroblast activation protein (FAP) and GPC3 simultaneously to address tumor diversity in HCC (58). The bispecific CAR-T cells displayed increased efficacy against tumor cells *in vitro*. Moreover, these bispecific CAR-T cells exhibited enhanced antitumor activity and significantly prolonged the survival of HCC mouse models, which represent a promising therapeutic approach to mitigate HCC recurrence (58).

### 3.2 AFP

AFP, a fetal-specific alpha-globulin produced during fetal development and detected in fetal blood and tissues, is also observable in the HCC tumors (59). In light of the fact that CAR-T cells specifically target tumor surface antigens rather than secreted or intracellular ones, Liu et al. engineered AFP-CAR-T cells capable of selectively binding to the AFP158-166 peptide presented by HLA-A02:01 on the surface of tumor cells *in vivo* (60). AFP-targeted CAR-T cells have demonstrated the capability to significantly inhibit tumor growth both *in vivo* and vitro (60).

### 3.3 c-Met

c-MET is a pro-oncogene responsible for encoding the receptor for hepatocyte growth factor (HGF) (61, 62). c-MET can trigger various downstream pathways, including the RAS/MAPK and phosphoinositide 3-kinase (PI3K)/AKT pathways, promoting tumor cell proliferation, growth and metastasis in HCC (63, 64). Jiang et al. developed the bispecific CAR-T cells that target both c-Met and programmed cell death ligand (PD-L1) and showed notable cytotoxicity against c-Met^+^PD-L1^+^ HCC cells (65). Furthermore, dual-targeted T cells exhibited potent inhibitory effects on tumorigenesis, surpassing the effects observed with single-targeted CAR-T cells (65). Recently, Qin and colleagues designed two MET-specific CARs: CD28ζ and 4-1BB (66). In comparison with MET inhibitors that targeted MET activation, MET-CAR-T cells recognized and eliminated HCC cells based on overall MET expression, with their activity being unrelated to MET signaling pathway activation (66). While MET-CAR.CD28ζ is favored for future advancements, optimizing the CAR construct design and implementing strategies to counteract CAR-T cell exhaustion induced by the tumor microenvironment are essential to enhance the therapeutic efficacy of MET-CAR-T cells (66).

### 3.4 NKG2D

NKG2D serves as a vital activating receptor present on NK cells and NKT cells (67). It recognizes and binds to a range of cell surface glycoproteins known as NKG2D ligands (NKG2DL), which are distantly related to MHC class I molecules (67). These ligands are upregulated in response to malignant transformation, thereby marking “stressed” or “harmful” cells for elimination by NKG2D^+^ lymphocytes (68, 69). The NKG2DL system offers a sophisticated immune surveillance mechanism that involves multiple layers of regulation to maintain a balance between early detection of stressed cells and prevention of autoimmunity induction (70).

In HCC, NKG2DL was elevated in tumor samples and related to aggressive carcinogenesis (71). Non-viral methods were employed in the generation of NKG2D CAR-T cells, involving the use of electroporation to introduce CAR-carrying piggyBac transposon plasmids, followed by *in vitro* expansion with K562 artificial antigen-presenting cells (72). This strategy not only preserved the anti-tumor capabilities of NKG2D CAR-T cells in laboratory settings but also led to a decrease in the levels of exhaustion markers typically found in T cells (72). Sun et al. performed research involving the development of NKG2D-BBz CAR-T cells utilizing the CAR derived from the extracellular domain of NKG2D, combining with 4-1BB and CD3ζ (73). These modified CAR-T cells exhibited potent cytotoxicity against HCC cells in laboratory settings and demonstrated therapeutic efficacy in xenograft models (73). The findings highlight the targeted eradication of HCC cells by NKG2D-BBz CAR-T cells in an NKG2DL-dependent manner, laying the groundwork for advancing towards clinical trials involving NKG2DL-positive patients (73).

Although the preliminary findings show promise, several potential drawbacks need to be taken into account. The monitoring of tumors by NKG2D can put significant pressure on their survival (74). Thus, it is not unexpected that certain human tumors release NKG2DL from their outer layer to escape immune attacks, leading to a rise in soluble NKG2DL levels (75). When the soluble NKG2D ligand binds, it can reduce the sensitivity of NKG2D in attacking cells throughout the body and weaken their ability to fight against tumors (76, 77). Moreover, evidence suggests that NKG2D plays a role in tumor formation in cases of inflammation-induced cancers including HCC (78). The impact of NKG2D CAR-T cells on either enhancing anti-tumor activity or promoting tumor-favorable inflammation in such scenarios is awaiting to be established.

### 3.5 CD133

Elevated CD133 is a common feature in HCC and is typically associated with an unfavorable prognosis for patients (79). The findings of a phase II clinical trial offered initial evidence that CD133-CAR-T cells exhibited significant anti-tumor effects and posed no significant safety risks in advanced HCC cases (80). The study revealed a median overall survival of 12 months and a median progression-free survival of 6.8 months, showcasing promising results in this advanced-stage cohort (80). Moreover, Yang et al. opted for a non-viral approach to effectively generate CD133-specific CAR-T cells capable of producing PD-1 scFv checkpoint inhibitors using an SB system derived from minicircle vectors, which has demonstrated reduced immunogenicity, lower costs, and enhanced safety in comparison to viral vectors (81). Thereafter, these engineered cells exerted significantly anti-tumor effects on HCC cells and xenograft mouse models, implying that employing an approach incorporating CD133 CAR-T and PD-1 scFv cells may present a viable choice for individuals dealing with advanced HCC and upregulated expression of CD133 (81). CoG133-CAR-T cells demonstrated significant transfection efficiency and displayed dual antigen-binding capabilities targeting CD133 and GPC3 (82). Extended survival and eradication of tumors were noted in HCC xenograft mice treated with CoG133-CAR-T cells, underscoring the significant promise of dual-specificity CAR-T cells (82).

### 3.6 EpCAM

Epithelial Cell Adhesion Molecule (EpCAM), a transmembrane protein located on the cell surface, has traditionally served as a primary indicator for carcinomas and is commonly employed in cancer diagnostics (83, 84). EpCAM expression related to poor prognosis in patients with advanced HCC (85). At present, EpCAM CAR-T cells are in the developmental stages for cancer therapy, with their potential application in HCC remaining unexplored (86). Multiple clinical trials are currently recruiting participants to assess the effectiveness and the safe profile of EpCAM CAR-T cells in individuals with advanced HCC (NCT02729493), postoperative relapse (NCT03013712), and refractory HCC (NCT05028933).

### 3.7 Other targets

Mucin 1 (MUC1) is overexpressed in various cancers and contributes to tumorigenesis in HCC (87, 88). At present, an ongoing clinical trial is investigating the use of MUC1 CAR-T cells for the treatment of HCC (NCT02587689). Additionally, increased levels of carcinoembryonic antigen (CEA) in serum have been identified as prognostic biomarkers in HCC (89). In the HITM-SIR trial, six patients with CEA-positive liver metastases were treated with CEA CAR-T cells and hepatic artery infusions combined with selective internal radiation therapy (90). Notably, there were no cases of severe adverse events observed throughout the trial, additional confirmation of the safety profile of CAR-T therapy was obtained (90, 91).

## 4 Challenges and prospects of CAR-T cell therapy

### 4.1 challenges with the inefficiency of CAR-T cell trafficking and infiltration

**Figure 2:**
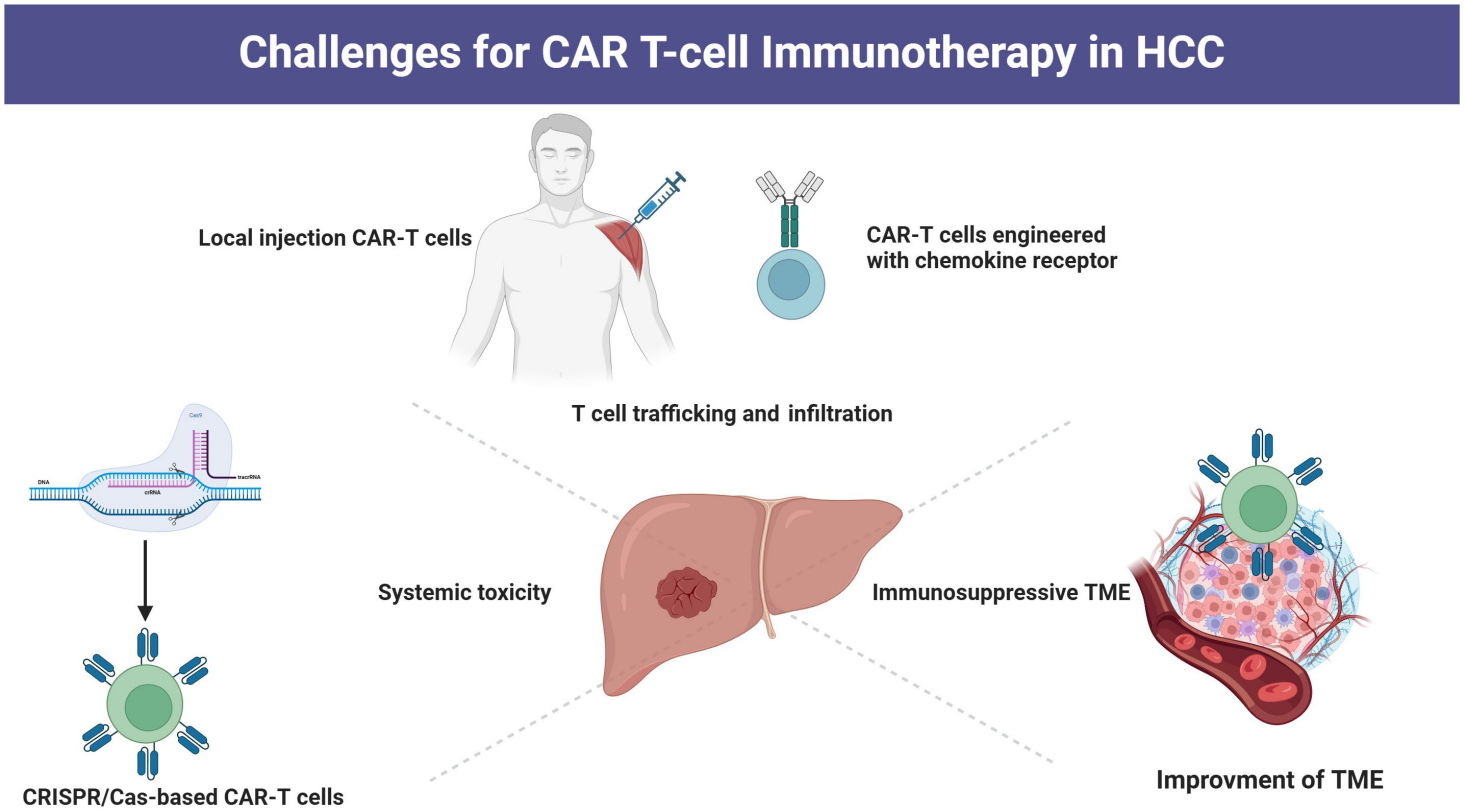
Strategies for overcoming challenges of CAR-T cell therapy.

Typically, CAR-T cells are administered via peripheral infusion, and their ability to migrate to the tumor site is essential for achieving cytolytic effects (92). However, T cells typically do not have the necessary chemokine receptors that play a key role in guiding T cells to tumor sites by interacting with chemokines released by tumor cells (93, 94). Moreover, in HCC tissue, there is a dense fibrotic structure that reduces the expression of chemokines, resulting in a significant decline in the ability of CAR-T cells to migrate and infiltrate the tumor (95). Under conditions of low oxygen levels, hypoxia-inducible factor-1 becomes activated and subsequently upregulates vascular endothelial growth factor, which interacts with receptors on endothelial cells (96). This process triggers the remodeling of the surrounding extracellular matrix and facilitates the development of irregular blood vessels within the tumor (96). These vessels exhibit structural abnormalities, including immature basement membranes, excessive branching, and discontinuous junctions. Such aberrant morphology contributes to increased permeability and suboptimal blood flow, ultimately impairing the transport of immune cells and therapeutic agents, thereby obstructing the penetration and homing of CAR T cells (97). Strategies to improve the trafficking and infiltration capabilities include the development of CAR-T cells with chemokine receptors and CAR-T cells engineered to express heparinase (98). Additionally, local administration of CAR-T cells has shown promising enhancements in the fight against tumors (99, 100) (**Figure 2**).

### 4.2 challenges with overcoming the immunosuppressive tumor microenvironment.

After CAR-T cells have effectively penetrated into a tumor, they encounter challenges presented by the hostile tumor microenvironment (TME) including hypoxia and low levels of nutrition (101, 102). Furthermore, the compact TME encircling HCC consists of various immunosuppressive cells including Treg cells, tumor-associated macrophages (TAMs), and fibroblasts, which can inhibit effective T cell responses by the secretion of immunosuppressive molecules and activating immune checkpoints (102, 103). For example, Luo et al. utilized a folate-targeted Toll-like receptor 7 agonist (FA-TLR7-1A) to specifically revitalize TAMs and myeloid-derived suppressor cells (MDSCs), transforming them from an immunosuppressive state to a pro-inflammatory phenotype, while maintaining the characteristics of other immune cell populations (104). The combination of FA-TLR7-1A with CAR-T cell therapy not only converted TAMs and MDSCs from an M2-like anti-inflammatory phenotype to an M1-like pro-inflammatory phenotype but also enhanced the infiltration and activation of both CAR-T cells and endogenous T cells within solid tumors, which markedly improved the effectiveness of standard CAR-T cell therapy against solid tumors in immunocompetent mice. Moreover, tumor glycosylation also plays a crucial role in inhibiting antitumor immune responses. Tumor glycans can impede the recognition of peptide epitopes by antibodies through steric hindrance (105). Furthermore, they foster an immunosuppressive environment by interacting with lectins present on immune cells (such as SIGLECand MGL) and by releasing galectins that bind to inhibitory molecules (including Galectin-9 and Galectin-3) (106, 107). Additionally, branched N-glycans can support interactions between immune checkpoints (like PD-1/PD-L1), thus increasing the activation threshold for T cell receptors (108). Therefore, it is important to explore gene editing targeting immune checkpoints on CAR-T cells, along with the use of targeted drugs to counteract the immunosuppressive TME and enhance metabolism programming, which could reduce the growth of HCC by improving cytotoxic T cell responses (32, 109, 110). Moreover, numerous strategies such as combining CAR-T therapy with immune checkpoint inhibitors (ICIs) or other immunostimulatory therapies, as well as engineering CAR-T cells to resist the immunosuppressive effects of cytokines have also been implemented to improve CAR-T therapy responses within TME (111, 112).

### 4.3 Challenges with systemic toxicity

Slight modifications in CAR design have been found to have significant impacts, not just on durability but also on safety (113). The infusion of CAR-T cells often leads to notable adverse events, such as off-target toxicity, cytokine release syndrome (CRS), and neurotoxicity (114, 115). Genome editing is increasingly contributing to enhancing the safety of CAR-T cells (32). TALENs and CRISPR–Cas nucleases have been utilized to target the granulocyte-macrophage colony-stimulating factor (GM-CSF) gene in CAR-T cells, aiming to inhibit GM-CSF secretion upon CAR-T cell activation, thereby potentially averting the activation of monocytes or macrophages, and subsequently reducing the risk of CRS (116, 117). Furthermore, structural alterations have shown promise in decreasing the toxicity of CAR-T cells while preserving their effectiveness in eradicating tumors (118).

## 5 Conclusion

CAR-T cells have emerged as a potentially revolutionary new strategy for the cancer treatment and have the potential to become a cornerstone of clinical management of HCC in the future. Despite the substantial advancements showcased by CAR-T cell therapy, there is still a considerable path ahead in CAR-T research to develop a practical treatment for HCC. To optimize CAR-T cell therapy in the future, further advancements should focus on enhancing CAR-T cell designs specific to HCC and reducing systematic toxicity. Sustained research endeavors focused on elucidating molecular mechanisms, refining treatment protocols, and overcoming therapeutic constraints are essential for driving the field forward toward achieving significant clinical results and ultimately enhancing the prognosis for patients with HCC.
